# Atrophic dermatofibrosarcoma protuberans: Two case reports and literature review

**DOI:** 10.3389/fonc.2023.1100398

**Published:** 2023-02-09

**Authors:** Yiting Li, Zile Chen, Shu Nie, Zhouwei Wu

**Affiliations:** Department of Dermatology, Shanghai General Hospital, Shanghai Jiao Tong University School of Medicine, Shanghai, China

**Keywords:** dermatofibrosarcoma protuberans, atrophic, molecular diagnosis, case report, literature review

## Abstract

Dermatofibrosarcoma protuberans is a rare, locally aggressive, slowly growing cutaneous fibroblastic sarcoma with a high recurrence rate and low metastatic potential. Atrophic dermatofibrosarcoma protuberans is a rare variant usually presents as atrophic plaques, easily neglected and misdiagnosed as benign lesions by patients and dermatologists. Here we report two cases of atrophic dermatofibrosarcoma protuberans, one of which was accompanied by pigment, and review other cases have been reported in the literature. Understanding the most up-to-date literature and early identification of these dermatofibrosarcoma protuberans variants can help clinicians avoid delayed diagnosis and improve prognosis.

## 1 Introduction

Dermatofibrosarcoma protuberans (DFSP) is a slowly growing, aggressive dermal soft tissue tumor with a high recurrence rate and low metastatic potential (1). Classic DFSP often presents as a typical protuberant nodule and is histologically characterized by monomorphic spindle cells arranged in a storiform pattern (2). Although DFSP is a rare sarcoma with an incidence of 0.8 to 4.2 cases per million persons per year, it is nevertheless one of the most common cutaneous sarcomas (3). Atrophic dermatofibrosarcoma protuberans is a histopathological variant of DFSP, which presents as an asymptomatic depressed plaque. Atypical clinical presentation and indolent behavior are easily neglected by patients and clinicians, leading to delayed diagnosis. In this report, we describe two cases of atrophic DFSP, one of which also with hyperpigmentation, and review the literature to improve our understanding of the clinical and histopathological features of this unusual variant.

## 2 Case reports

### 2.1 Case 1

**Figure 1:**
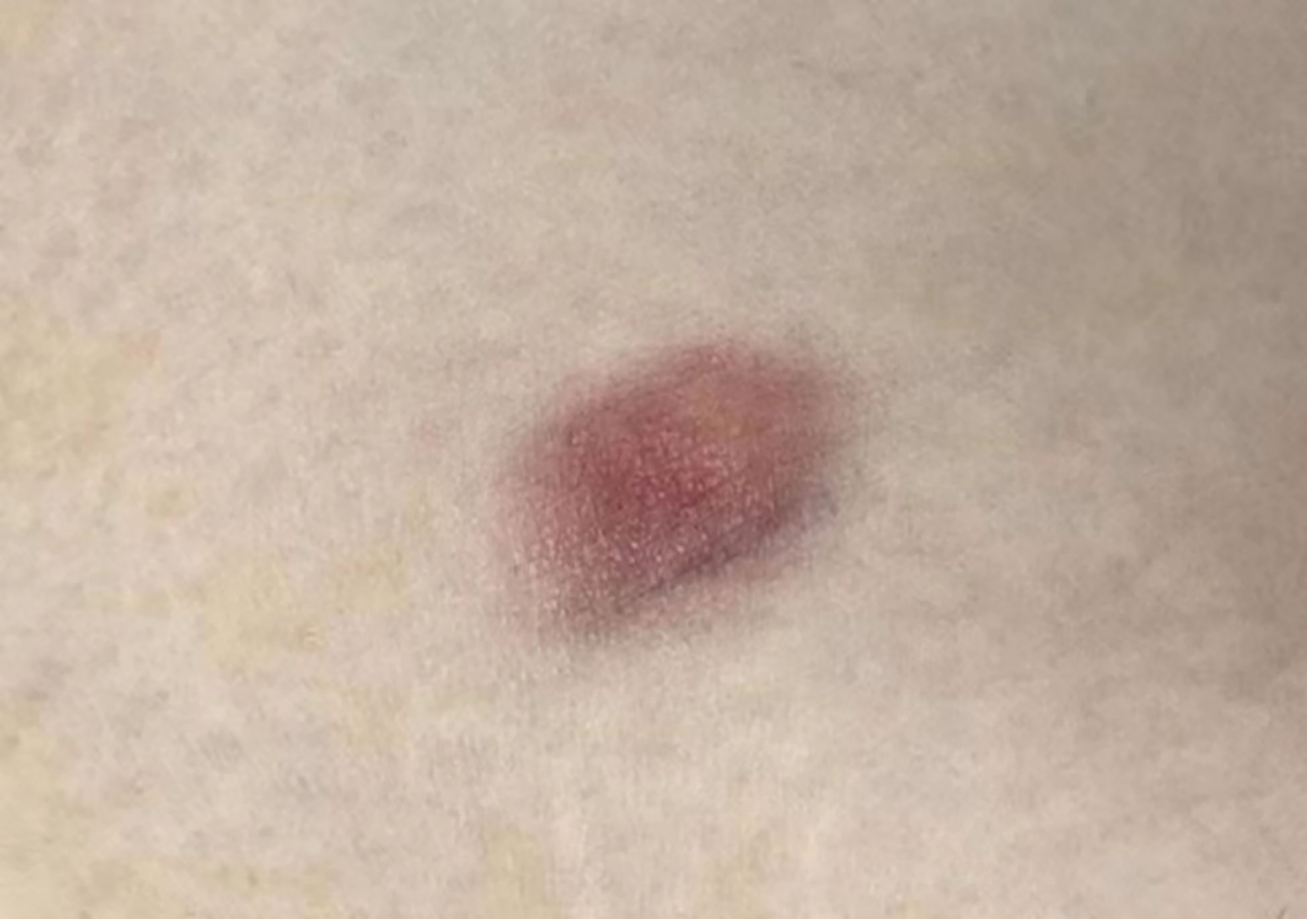
A 16×10mm atrophic erythematous plaque on her right chest.

**Figure 2:**
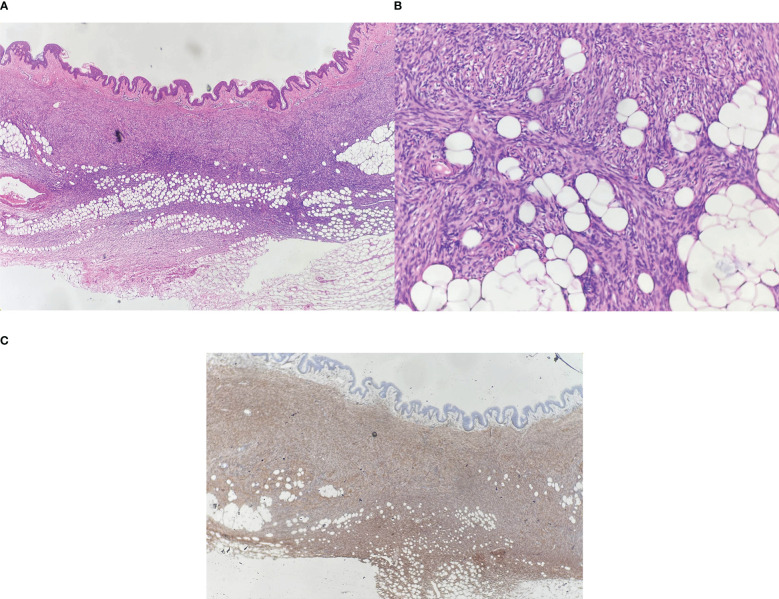
**(A)** Spindle cells infiltrate into the deep dermis and subcutaneous tissue, Hematoxylin-eosin (HE) ×40; **(B)** Dense proliferation of spindle cells in a storiform pattern, HE ×100; **(C)** Spindle cells are strongly positive for CD34, ×40.

A 31-year-old woman attended our dermatology department to evaluate an erythematous plaque on her right chest that had been slowly progressing for four years. She was diagnosed as atrophic scar previously. Physical examination showed a 16×10 mm asymptomatic, smooth, atrophic plaque (**Figure 1**). Histopathologic examination revealed a monomorphic fibrohistiocytic spindle cell tumor arranged in a storiform pattern infiltrating the deep dermis and subcutaneous tissue forming honeycomb-like pattern (**Figures 2A, B**). Immunohistochemical analysis showed that cells were prominently positive for CD34 (**Figure 2C**) and negative for protein S-100. Next-generation sequencing (NGS) detected a fusion between exon 5 of COL1A1 and exon 2 of PDGFB. Based on these findings, the lesion was diagnosed as atrophic DFSP. The patient was treated by Mohs micrographic surgery and no recurrence was observed at the 1-year follow-up.

### 2.2 Case 2

**Figure 3:**
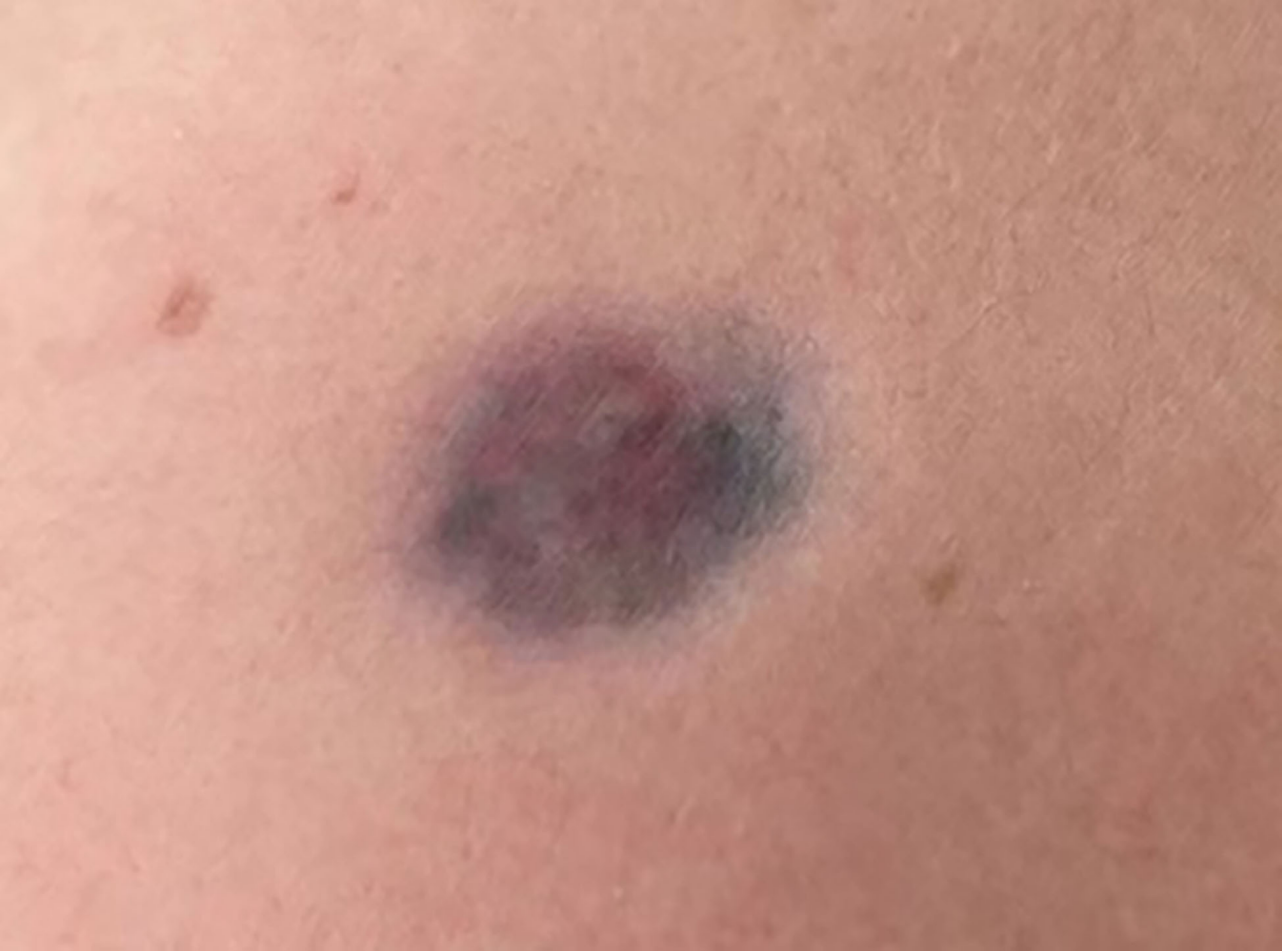
A round erythematous-to-bluish atrophic plaque.

**Figure 4:**
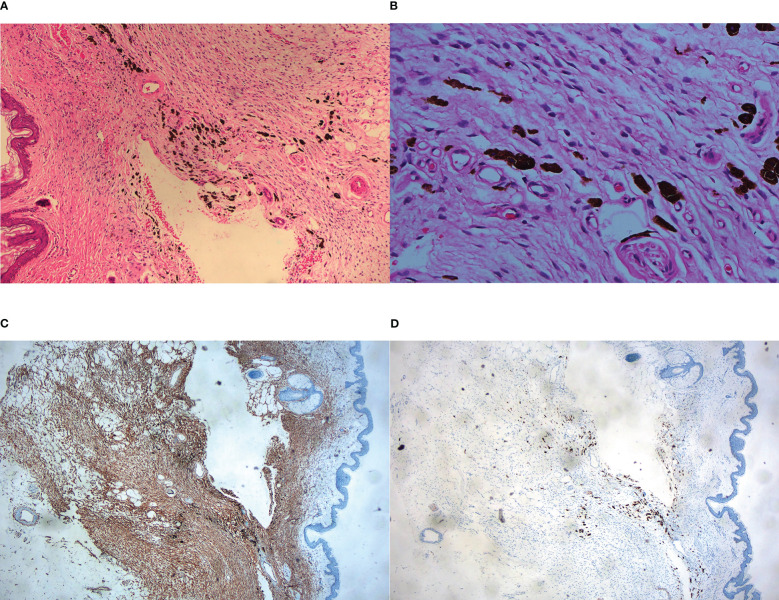
Histopathological examination. **(A)** Fascicles of spindle cells with reduced dermal thickness by about half on low-power view, HE ×40; **(B)** Monomorphic spindle cells with bland cytoplasm and scattered dendritic cells containing abundant melanin, HE ×200; **(C)** Immunohistochemistry showed CD34^+^ cells infiltrating into subcutis and forming honeycomb-like pattern, ×40; **(D)** Immunohistochemistry showed spindle cells were negative for S-100, in contrast, melanin-bearing dendritic cells were positive for S-100, ×100.

A 45-year-old man presented to our dermatology department with a 5-year history of a depressed plaque on his left shoulder. The lesion was asymptomatic and slowly enlarged. He was previously diagnosed as blue nevus and anetoderma in other clinics. Physical examination showed a 30×20 mm round erythematous-to-bluish plaque with atrophy (**Figure 3**). Histopathologic analysis revealed infiltration of spindle cells with slender wavy nuclei into the deep dermis, arranged in parallel or horizontally oriented fascicles. Cells containing brown pigment were scattered in the dermis (**Figures 4A, B**). Immunohistochemical staining showed that the spindle cells were diffusely positive for CD34 but negative for S-100 while melanin-laden dendritic cells were positive for S-100 (**Figures 4C, D**). NGS detected a fusion between exon 46 of COL1A1 and exon 2 of PDGFB. Based on the clinicopathologic correlation, a diagnosis of atrophic pigmented DFSP was made. Mohs micrographic surgery was performed to excise the lesion.

## 3 Discussion

Atrophic dermatofibrosarcoma protuberans is a rare variant accounting for 1.7% of all DFSP cases (4). It was first described by Lambert in 1985 (5). Unlike the classical DFSP, atrophic DFSP usually presents as a slow-growing, atrophic or sclerotic plaque, which is more easily misdiagnosed as morphea, sclerodermiform basal cell carcinoma, atrophic scar, scleroderma, anetoderma, atrophic dermatofibroma, resolving panniculitis and lipoatrophy (6). Histologically, in addition to the storiform pattern noted in classical DFSP, neoplastic spindle cells arranged in parallel or horizontally oriented fascicles are also seen in atrophic DFSP (4). On immunohistochemistry, all tumors showed diffuse and strong positivity of CD34. Due to the overlap of morphology and immunoprofile, atrophic DFSP should be differentiated from other spindle cell tumors, including dermatofibroma, solitary fibrous tumor, spindle cell/pleomorphic lipomas, neurofibroma, superficial CD34-positive fibroblastic tumor, and medallion-like dermal dendrocyte hamartoma (7).

Molecular analysis is used to detect the COL1A1-PDGFB fusion gene, which is specifically expressed in DFSP and not related to pathological subtypes. FISH and NGS are two main molecular techniques for detecting the fusion gene. FISH is a straightforward method performed with either fusion probes or break-apart probes, which has good sensitivity and specificity. However, it doesn’t provide detailed breakpoint information on both translocation partners. NGS is another technique for detecting fusion transcripts. Previous studies have shown that NGS was more sensitive than FISH for the COL1A1-PDGFB fusion detection, especially in borderline cases (8). Additionally, NGS can effectively detect all fusion genes and fusion partners, which can make up for the shortcomings of conventional detection methods, such as missed detection, false negatives, and the inability to distinguish fusion partners. However, there are also some challenges with data analysis and interpretation, analytical validation, high-quality sample tissues, and high testing costs (9).

Surgical excision is the gold standard for the primary treatment of DFSP with wide excision and margin-controlled removal (i.e. Mohs and slow Mohs) as viable options (10, 11). For cases with recurrent, unresectable, and metastatic tumors, radiation therapy and targeted immunotherapy such as tyrosine kinase inhibitors have been shown to be effective (12). Imatinib mesylate can competitively inhibit ATP binding to the PDGF-β receptor, which slows down kinase activity, limits the growth of the tumor, and promotes apoptosis (13).

Till now, 91 cases with atrophic DFSP, including ours, have been reported (**Table 1**). The age ranges from 7 months to 72 years with an average of 26.7. It seems like the atrophic variant most frequently occurs during the second to fifth decades of life (n=65), which is younger compared with classic DFSP. Children and congenital cases account for 34% of all cases (n=31). In contrast to classic DFSP has no sex bias (10), atrophic DFSP shows a preference for females with a F:M ratio of 3:1. Trunk and extremities are the most frequently involved sites (n=87), which are consistent with classic DFSP (1). The tumor size ranges from 0.5 to 13cm in greatest diameter. Clinical diagnosis includes morphea, morpheaform basal cell carcinoma, anetoderma, lymphocytoma, lipoatrophy, atrophic scar, and neurofibroma. Delayed diagnosis and misdiagnosis are common. 5/8 cases of atrophic pigmented DFSP were misdiagnosed as benign lesions, including nevus of Ota, postinflammatory hyperpigmentation, hemangioma, and lipoatrophy (20, 55, 61). A high index of suspicion and a broad differential diagnosis by dermatologists is necessary.

**Table 1:** Reported cases of atrophic dermatofibrosarcoma protuberans.

Case (No.)	Age(y)	Sex	Site	Size(cm)	Clinical diagnosis	Reference
1	15	M	Supraclavicular	5×7	Morphea, morpheaform BCC	(5)
2	50	F	Inferior aspect of abdomen	3×6	DFSP	(5)
3	37	F	Chest	5×8	Morphea, morpheaform BCC	(5)
4	15	M	Posterior aspect of shoulder	3×5	Morphea	(5)
5	28	M	Inferior aspect back	2.5	N/S	(5)
6	25	F	Subclavicular	2×3	BCC	(14)
7	21	F	Upper aspect of back	1×1.5	Anetoderma	(15)
8	27	F	Upper aspect of back	2×3	Sclerosing BCC	(15)
9	7	F	Upper Arm	N/S	DFSP	(16)
10	1	M	Lower Back	N/S	DFSP	(16)
11	12	F	Back	N/S	DFSP	(16)
12	9	F	Shoulder	N/S	DFSP	(16)
13	6	M	Right shoulder	N/S	DFSP	(16)
14	5	M	Back	N/S	DFSP	(16)
15	10	F	Buttock	N/S	DFSP	(16)
16	10	F	Forefoot	N/S	DFSP	(16)
17	25	F	Shoulder	2.5×1.5	N/S	(17)
18	16	F	Left periumbilical region	3×4	Congenital atrophic DFSP	(18)
19	40	F	Periumbilical	N/S	Lymphocytoma	(19)
20	55	F	Shoulder	3×6	DFSP, lymphocytoma	(19)
21	42	F	Groin	N/S	BCC, scar	(19)
22	24	F	Infraorbital	N/S	Nevus of Ota	(20)
23	1.5	M	Left ankle	3.5×6.5	DFSP	(21)
24	42	M	Shoulder	2×3	N/S	(22)
25	21	F	Subclavicular	2×3	Anetoderma	(23)
26	53	M	Left clavicle	7×5	N/S	(24)
27	13	M	Left calf	4	N/S	(25)
28	3	F	Right thigh	0.5	Morphea	(25)
29	16	F	Left calf	3	Atrophic plaque	(25)
30	1.5	M	Left ankle	N/S	Atrophoderma, lipoatrophy	(25)
31	3	F	Periumbilical	5×4	Morphea	(25)
32	21	M	Epigastric	N/S	DFSP	(25)
33	72	F	Midback	3×1.5	N/S	(26)
34	16	F	Right leg	6×8	N/S	(27)
35	40	M	Chest	6	DFSP	(28)
36	26	F	Right breast	1×2	Atrophic DFSP	(29)
37	65	M	Lower aspect of back	2.5×3	Anetoderma, atrophic scar	(30)
38	41	F	Right Chest	4×5	Atrophic DFSP	(31)
39	23	M	Left anterior shoulder	3.3 × 2.8	Atrophic DFSP	(32)
40	16	F	Right thigh	N/S	DFSP	(33)
41	48	F	Left upper abdomen	2×0.8	Atrophic DFSP	(34)
42	55	F	Epigastric region	2×1.5	Atrophic DFSP	(35)
43	29	F	Right thigh	10	Atrophic DFSP	(36)
44	36	M	Left cheek	4×4	DFSP	(37)
45	30	M	Mid-back	5×3	Atrophic DFSP	(38)
46	7mo	M	Left upper groin	1.6×0.7	Atrophic DFSP	(39)
47	14	F	Right leg	4×3	Atrophic DFSP	(40)
48	18	F	Left supraclavicular	2	Atrophic DFSP	(41)
49	14	M	Hand	2.5	Congenital atrophic DFSP	(41)
50	8	F	Right thigh	2.5×2	Congenital atrophic DFSP	(41)
51	52	F	Left buttock	6 x 5	Atrophic DFSP	(42)
52	40	F	Right pars lumbalis	N/S	Atrophic DFSP	(43)
53	30	F	Upper abdomen	6×5	Atrophic DFSP	(44)
54	5	M	Right buttock	N/S	Congenital DFSP	(45)
55	36	M	Left chest	8×10	Morphea	(46)
56	30	F	Left Waist	1.3×0.8	Atrophic DFSP	(47)
57	37	F	Left shoulder	1×1.5	Atrophic DFSP	(48)
58	23	F	Back	2	Atrophic DFSP	(49)
59	24	F	Chest	N/S	Atrophic DFSP	(50)
60	19	F	Right precordium	2.5×3	Congenital atrophic DFSP	(51)
61	34	F	Left buttock	1.1×1.2	Atrophic pigmented DFSP	(52)
62	6	F	Right lower abdomen	4×2.5	Atrophic DFSP	(53)
63	15	M	Back	N/S	Atrophic DFSP	(54)
64	7	F	Left wrist	2×4	Lipoatrophy	(55)
65	8	F	Left forearm	1×1	Hemangioma	(55)
66	48	F	Anterior chest wall	13×12	Atrophic DFSP	(56)
67	31	F	Left lower abdominal wall	1.5	DFSP	(4)
68	14	M	Right upper abdominal wall	2	Spindle cell neoplasm, suspicious of DFSP	(4)
69	7	M	Left forearm	0.5	Atrophic pigmented DFSP	(4)
70	45	F	Infraclavicular region	N/S	DFSP	(4)
71	23	F	Left chest wall	1.5	Neurofibroma	(4)
72	44	M	Right back	2.5	Neurofibroma	(4)
73	27	F	Shoulder	2	Possible DFSP	(4)
74	19	M	Right chest wall	2.5	DFSP	(4)
75	28	F	Infraclavicular region	1	Neurofibroma	(4)
76	28	F	Chest wall	1.5	DFSP	(4)
77	24	F	Left lower neck	2	Spindle cell neoplasm	(4)
78	48	F	Left cheek	1.4	Spindle cell neoplasm	(4)
79	47	F	Left chest wall	1	Spindle cell neoplasm	(4)
80	34	M	Back	1.2	Spindle cell neoplasm, suspicious of DFSP	(4)
81	36	F	Left groin	2	Spindle cell neoplasm	(4)
82	63	F	Chest wall	1	Cutaneous diffuse neurofibroma	(4)
83	30	F	Chest	4×2	Atrophic DFSP	(57)
84	N/S	F	Right inferior breast	4×3.5	Atrophic DFSP	(58)
85	19	F	Right chest	12×5	Congenital atrophic DFSP	(59)
86	26	M	Left back	3×2.5	Atrophic pigmented DFSP	(60)
87	33	F	Left upper back	1.6 × 1.3	Postinflammatory hyperpigmentation	(61)
88	39	F	Right lower leg	N/S	Dermatofibroma	(62)
89	39	F	Abdomen	N/S	Morphea	(63)
90	31	F	Right chest	1.6×1	Atrophic DFSP	Our case
91	45	M	Left shoulder	N/S	Atrophic pigmented DFSP	Our case

BCC, Basal cell carcinoma; DFSP, dermatofibrosarcoma protuberans; F, female; M, male; N/S, not stated.

In summary, atrophic DFSP is a diagnostic challenge to both clinicians and pathologists due to its atypical clinical presentation. Because of the initial benign and indolent behavior, many patients only seek medical care years after its onset. Here, we present a case series to further characterize its clinical and pathological features and enhance recognition of atrophic DFSP. As with these cases, atrophic lesions with no apparent cause and no symptoms should be aware of atrophic DFSP in the early stage. Histopathologic, immunohistochemical, and molecular examinations are necessary to help reduce misdiagnosis and improve prognosis.

## Data availability statement

The original contributions presented in the study are included in the article/supplementary material. Further inquiries can be directed to the corresponding author.

## Author contributions

YL analyzed the clinical data and drafted the manuscript. ZC collected the data and reviewed literature. SN managed the patient. ZW designed and revised the manuscript. All authors contributed to the article and approved the submitted version.

## References

[1] Allen A Ahn C Sangüeza OP . Dermatofibrosarcoma protuberans. Dermatol Clin (2019) 37(4):483–8. doi: 10.1016/j.det.2019.05.006 31466588

[2] Mujtaba B Wang F Taher A Aslam R Madewell JE Spear R . Dermatofibrosarcoma protuberans: Pathological and imaging review. Curr Probl Diagn Radiol (2021) 50(2):236–40. doi: 10.1067/j.cpradiol.2020.05.011 32620358

[3] Kohlmeyer J Steimle-Grauer SA Hein R . Cutaneous sarcomas. J Dtsch Dermatol Ges (2017) 15(6):630–48. doi: 10.1111/ddg.13249 28591457

[4] Xu S Zhao L Wang J . Atrophic dermatofibrosarcoma protuberans: a clinicopathological study of 16 cases. Pathology (2019) 51(6):615–20. doi: 10.1016/j.pathol.2019.06.002 31447095

[5] Lambert WC Abramovits W Gonzalez-Sevra A Souchon E Schwartz RA Little WP Jr. Dermatofibrosarcoma non-protuberans: description and report of five cases of a morpheaform variant of dermatofibrosarcoma. J Surg Oncol (1985) 28(1):7–11. doi: 10.1002/jso.2930280104 2578589

[6] Acosta AE Vélez CS . Dermatofibrosarcoma protuberans. Curr Treat Options Oncol (2017) 18(9):56. doi: 10.1007/s11864-017-0498-5 28795284

[7] Thway K Noujaim J Jones RL Fisher C . Dermatofibrosarcoma protuberans: pathology, genetics, and potential therapeutic strategies. Ann Diagn Pathol (2016) 25:64–71. doi: 10.1016/j.anndiagpath.2016.09.013 27806849

[8] Zhu R Yan J Li B Tan F Yan W Shen J . Determination of COL1A1-PDGFB breakpoints by next-generation sequencing in the molecular diagnosis of dermatofibrosarcoma protuberans. Exp Mol Pathol (2021) 122:104672. doi: 10.1016/j.yexmp.2021.104672 34371012

[9] Cheng YW Meyer A Jakubowski MA Keenan SO Brock JE Azzato EM . Gene fusion identification using anchor-based multiplex PCR and next-generation sequencing. J Appl Lab Med (2021) 6(4):917–30. doi: 10.1093/jalm/jfaa230 33537766

[10] Saiag P Grob J-J Lebbe C Malvehy J del Marmol V Pehamberger H . Diagnosis and treatment of dermatofibrosarcoma protuberans. European consensus-based interdisciplinary guideline. Eur J Cancer (2015) 51(17):2604–8. doi: 10.1016/j.ejca.2015.06.108 26189684

[11] Durack A Gran S Gardiner MD Jain A Craythorne E Proby CM . A 10-year review of surgical management of dermatofibrosarcoma protuberans. Br J Dermatol (2021) 184(4):731–9. doi: 10.1111/bjd.19346 32599647

[12] Badhey AK Tikhtman R Tang AL . Management of dermatofibrosarcoma protuberans. Curr Opin Otolaryngol Head Neck Surg (2021) 29(4):278–82. doi: 10.1097/MOO.0000000000000721 33993132

[13] Rutkowski P Van Glabbeke M Rankin CJ Ruka W Rubin BP Debiec-Rychter M . Imatinib mesylate in advanced dermatofibrosarcoma protuberans: pooled analysis of two phase II clinical trials. J Clin Oncol (2010) 28(10):1772–9. doi: 10.1200/JCO.2009.25.7899 PMC3040044 20194851

[14] Sei Y Kimura A Kobayashi H Ishikura N Yasuda Y . Dermatofibrosarcoma protuberans representing a depressed lesion. Skin Res (1986) 28(4):638–43. doi: 10.11340/SKINRESEARCH1959.28.638

[15] Page EH Assaad DM . Atrophic dermatofibroma and dermatofibrosarcoma protuberans. J Am Acad Dermatol (1987) 17(6):947–50. doi: 10.1016/S0190-9622(87)70283-7 3429722

[16] McKee PH Fletcher CD . Dermatofibrosarcoma protuberans presenting in infancy and childhood. J Cutan Pathol (1991) 18(4):241–6. doi: 10.1111/j.1600-0560.1991.tb01230.x 1939782

[17] Ashack RJ Tejada E Parker C Hanke CW . A localized atrophic plaque on the back. dermatofibrosarcoma protuberans (DFSP) (Atrophic variant). Arch Dermatol (1992) 128(4):549, 52. doi: 10.1001/archderm.1992.01680140133020 1580667

[18] Annessi G Cimitan A Girolomoni G Giannetti A . Congenital dermatofibrosarcoma protuberans. Pediatr Dermatol (1993) 10(1):40–2. doi: 10.1111/j.1525-1470.1993.tb00011.x 8493166

[19] Zelger BW Ofner D Zelger BG . Atrophic variants of dermatofibroma and dermatofibrosarcoma protuberans. Histopathology (1995) 26(6):519–27. doi: 10.1111/j.1365-2559.1995.tb00270.x 7545142

[20] Chuan MT Tsai TF Wu MC Wong TH . Atrophic pigmented dermatofibrosarcoma presenting as infraorbital hyperpigmentation. Dermatology (1997) 194(1):65–7. doi: 10.1159/000246061 9031796

[21] Bouyssou-Gauthier ML Labrousse F Longis B Bedane C Bernard P Bonnetblanc JM . Dermatofibrosarcoma protuberans in childhood. Pediatr Dermatol (1997) 14(6):463–5. doi: 10.1111/j.1525-1470.1997.tb00691.x 9436846

[22] Kobayashi T Hasegawa Y Konohana A Nakamura N . A case of bednar tumor. immunohistochemical positivity for CD34. Dermatology (1997) 195(1):57–9. doi: 10.1159/000245689 9267742

[23] Fujimoto M Kikuchi K Okochi H Furue M . Atrophic dermatofibrosarcoma protuberans: a case report and review of the literature. Dermatology (1998) 196(4):422–4. doi: 10.1159/000017936 9669119

[24] Davis DA Sánchez RL . Atrophic and plaquelike dermatofibrosarcoma protuberans. Am J dermatopathol (1998) 20(5):498–501. doi: 10.1097/00000372-199810000-00014 9790114

[25] Martin L Combemale P Dupin M Chouvet B Kanitakis J Bouyssou-Gauthier ML . The atrophic variant of dermatofibrosarcoma protuberans in childhood: a report of six cases. Br J Dermatol (1998) 139(4):719–25. doi: 10.1046/j.1365-2133.1998.02476.x 10025975

[26] See AC Kossard SS Murrell DF . Guess what. dermatofibrosarcoma protuberans presenting as an atrophic red plaque. Eur J Dermatol (2001) 11(2):147–9. 11275815

[27] Marini M Saponaro A Magariños G de Baldrich A Lynch P Remorino L . Congenital atrophic dermatofibrosarcoma protuberans. Int J Dermatol (2001) 40(7):448–50. doi: 10.1046/j.1365-4362.2001.01217.x 11679000

[28] Teixeira F Devlin M Hung N Yun K . An atrophic plaque on the chest. Aust Fam Physician (2002) 31(4):359–60. 12043129

[29] Cavuşoğlu T Yavuzer R Tuncer S . Dermatofibrosarcoma protuberans of the breast. Aesthetic Plast Surg (2003) 27(2):104–6. doi: 10.1007/s00266-003-0107-9 14629060

[30] Young CR 3rd Albertini MJ . Atrophic dermatofibrosarcoma protuberans: case report, review, and proposed molecular mechanisms. J Am Acad Dermatol (2003) 49(4):761–4. doi: 10.1067/S0190-9622(03)00793-X 14512938

[31] Sheehan DJ Madkan V Strickling WA Peterson CM . Atrophic dermatofibrosarcoma protuberans: a case report and reappraisal of the literature. Cutis (2004) 74(4):237–42. 15551716

[32] Wu JK Malik MM Egan CA . Atrophic dermatofibrosarcoma protuberans: an uncommon and misleading variant. Australas J Dermatol (2004) 45(3):175–7. doi: 10.1111/j.1440-0960.2004.00083.x 15250897

[33] Sathyanarayana BD . Childhood onset dermatofibrosarcoma protuberans. Indian J Dermatol Venereol Leprol (2004) 70(5):310–2. 17642647

[34] Sinovich V Hollowood K Burge S . Atrophic dermatofibrosarcoma protuberans. Australas J Dermatol (2005) 46(2):114–7. doi: 10.1111/j.1440-0960.2005.00156.x 15842408

[35] Hirashima N Misago N Shinogi T Inoue T Miura Y Narisawa Y . Atrophic dermatofibrosarcoma protuberans with diffuse eosinophilic infiltrate. J Dermatol (2006) 33(7):486–8. doi: 10.1111/j.1346-8138.2006.00114.x 16848822

[36] Kostrzewa E Beylot-Barry M Vergier B Pedeutour F Beylot C . [Childhood-onset multifocal atrophic dermatofibrosarcoma]. Ann Dermatol Venereol (2006) 133(4):359–61. doi: 10.1016/S0151-9638(06)70915-2 16733451

[37] Hanabusa M Kamo R Harada T Ishii M . Dermatofibrosarcoma protuberans with atrophic appearance at early stage of the tumor. J Dermatol (2007) 34(5):336–9. doi: 10.1111/j.1346-8138.2007.00283.x 17408444

[38] Llombart B Sanmartin O Requena C Monteagudo C Botella-Estrada R Nagore E . Atrophic dermatofibrosarcoma protuberans with the fusion gene COL1A1-PDGFB. J Eur Acad Dermatol Venereol (2008) 22(3):371–4. doi: 10.1111/j.1468-3083.2007.02327.x 18005119

[39] Feramisco J Larsen F Weitzul S Cockerell C Ghali F . Congenital atrophic dermatofibrosarcoma protuberans in a 7-month-old boy treated with mohs micrographic surgery. Pediatr Dermatol (2008) 25(4):455–9. doi: 10.1111/j.1525-1470.2008.00718.x 18789087

[40] Gerlini G Mariotti G Urso C Brandani P Reali UM Borgognoni L . Dermatofibrosarcoma protuberans in childhood: two case reports and review of the literature. Pediatr Hematol Oncol (2008) 25(6):559–66. doi: 10.1080/08880010802235066 18728975

[41] Marque M Bessis D Pedeutour F Viseux V Guillot B Fraitag-Spinner S . Medallion-like dermal dendrocyte hamartoma: the main diagnostic pitfall is congenital atrophic dermatofibrosarcoma. Br J Dermatol (2009) 160(1):190–3. doi: 10.1111/j.1365-2133.2008.08896.x 19016705

[42] Bakry O Attia A . Atrophic dermatofibrosarcoma protuberans. J Dermatol Case Rep (2012) 6(1):14–7. doi: 10.3315/jdcr.2012.1089 PMC3322104 22514584

[43] Qiao J Patel KU López-Terrada D Fang H . Atrophic dermatofibrosarcoma protuberans: report of a case demonstrated by detecting COL1A1-PDGFB rearrangement. Diagn Pathol (2012) 7:166. doi: 10.1186/1746-1596-7-166 23199263 PMC3539889

[44] Al-Balbeesi AO . Atrophic dermatofibrosarcoma protuberans and enlargement with pregnancy: Case report and literature review. J Saudi Soc Dermatol Dermatol Surg (2012) 16:21–3. doi: 10.1016/j.jssdds.2011.09.001

[45] de Morais OO de Araújo LC Gomes CM Coutinho AS Souza FA Costa IM . Congenital dermatofibrosarcoma protuberans. Cutis (2012) 90(6):285–8. 23409476

[46] Wen P Yu R Wang L . Atrophic dermatofibrosarcoma protuberans: a case report. Int J Dermatol (2013) 52(4):463–5. doi: 10.1111/j.1365-4632.2011.05309.x 23113648

[47] Han XY Wei HQ Pan Q Liu J . [Atrophic dermatofibrosarcoma protuberans: report of a case]. Zhonghua Bing Li Xue Za Zhi (2013) 42(1):52–3. doi: 10.3760/cma.j.issn.0529-5807.2013.01.014 23611277

[48] Güngör S Büyükbabani N Büyük M Tarıkçı N Kocatürk E . Atrophic dermatofibrosarcoma protuberans: are there specific dermatoscopic features? J Dtsch Dermatol Ges (2014) 12(5):425–7. doi: 10.1111/ddg.12308 24797753

[49] Akay BN Unlu E Erdem C Heper AO . Dermatoscopic findings of atrophic dermatofibrosarcoma protuberans. Dermatol Pract Concept (2015) 5(1):71–3. doi: 10.5826/dpc.0501a12 PMC4325695 25692086

[50] Xu WJ Wang JS . Atrophic dermatofibrosarcoma protuberans with the fusion gene COL1A1-PDGFB detected by RT-PCR using only a single primer pair. Int J Clin Exp Pathol (2015) 8(6):7457–63. PMC4525987 26261653

[51] Makino M Sasaoka S Nakanishi G Makino E Fujimoto W . Congenital atrophic dermatofibrosarcoma protuberans detected by COL1A1-PDGFB rearrangement. Diagn Pathol (2016) 11:24. doi: 10.1186/s13000-016-0474-6 26932148 PMC4774026

[52] Taura M Wada M Kataoka Y Ueda Y Takenaka H Katoh N . Case of pigmented dermatofibrosarcoma protuberans with atrophic change. J Dermatol (2016) 43(10):1231–2. doi: 10.1111/1346-8138.13366 27029004

[53] Liu Y Zhang B Han X Ma L . Pediatric atrophic dermatofibrosarcoma protuberans. Pediatr Investig (2017) 1(1):50–2. doi: 10.1002/ped4.12008 PMC7331386 32851219

[54] Aragão S Leite E Cardoso AEC Houly RLS . An unusual variant of atrophic dermatofibrosarcoma protuberans. Bras Dermatol (2018) 93(2):282–4. doi: 10.1590/abd1806-4841.20187049 PMC5916409 29723383

[55] Zhang Y Chen H Sun J . Two childhood cases of pigmented dermatofibrosarcoma protuberans with atrophic change. Eur J Dermatol (2018) 28(2):225–6. doi: 10.1684/ejd.2017.3198 29336320

[56] Saigusa R Miyagawa T Toyama S Omatsu J Miyagaki T Masui Y . Dermatofibrosarcoma protuberans presenting as a Large atrophic plaque on the chest. Acta Derm Venereol (2018) 98(1):155–6. doi: 10.2340/00015555-2800 28929166

[57] Fagan KK Sanchez AT Davis LS . Childhood onset atrophic plaque on the chest of a woman with history of acral lentiginous melanoma. Int J Dermatol (2019) 58(6):669–71. doi: 10.1111/ijd.14376 30609013

[58] Kibbi N Wang D Wang WL Galan A Leffell DJ Christensen SR . Dermatofibrosarcoma protuberans in pregnancy: a case series and review of the literature. Int J Dermatol (2021) 60(9):1114–9. doi: 10.1111/ijd.15497 33818755

[59] Chow ML Sennett R Hinds B Brian Jiang SI . Large Atrophic plaque on the chest: Answer. Am J dermatopathol (2021) 43(4):308–9. doi: 10.1097/DAD.0000000000001791 33742999

[60] Bai J Liu B Liu T Qiao J Fang H . Atrophic pigmented dermatofibrosarcoma protuberans: A case report and literature review. Front Oncol (2021) 11:669754. doi: 10.3389/fonc.2021.669754 34221984 PMC8245761

[61] Lin P Yang Z Tu P Li H . Atrophic pigmented dermatofibrosarcoma protuberans misdiagnosed as hyperpigmentation. Indian J Dermatol Venereol Leprol (2021) 87(5):693–5. doi: 10.25259/IJDVL_713_20 34379942

[62] Wang P Xiong JX Chen AJ Cai T . A case of atrophic dermatofibrosarcoma protuberans. Ann Dermatol (2022) 34(5):387–8. doi: 10.5021/ad.20.144 PMC9561293 36198632

[63] Crnarić I Šitum M Delaš Aždajić M Vučić M Buljan M . From morphea to dermatofibrosarcoma protuberans. Acta Dermatovenerol Croat (2022) 30(2):113–5. 36254545

